# Vaccine-Derived Polioviruses and Children with Primary Immunodeficiency, Iran, 1995–2014

**DOI:** 10.3201/eid2210.151071

**Published:** 2016-10

**Authors:** Mohammadreza Shaghaghi, Shohreh Shahmahmoodi, Hassan Abolhassani, Saeed Soleyman-jahi, Leila Parvaneh, Sussan Mahmoudi, Zahra Chavoshzadeh, Reza Yazdani, Seyed Mohsen Zahraei, Mohsen Ebrahimi, Mohammad H. Eslamian, Hamideh Tabatabaie, Maryam Yousefi, Yaghoob M. Kandelousi, Aliasghar Oujaghlou, Nima Rezaei, Asghar Aghamohammadi

**Affiliations:** Tehran University of Medical Sciences, Tehran, Iran (M. Shaghaghi, S. Shahmahmoodi, H. Abolhassani, S. Soleyman-jahi, L. Parvaneh, M. Ebrahimi, M.H. Eslamian, N. Rezaei, A. Aghamohammadi);; Universal Scientific Education and Research Network, Tehran (M. Shaghaghi, H. Abolhassani, S. Soleyman-jahi, R. Yazdani, N. Rezaei, A. Aghamohammadi);; Research Center for Immunodeficiences, Tehran (M. Shaghaghi, H. Abolhassani, N. Rezaei, A. Aghamohammadi);; Tehran University of Medical Sciences School of Public Health, Tehran (S. Shahmahmoodi, H. Tabatabaie, M. Yousefi, Y.M. Kandelousi, A. Oujaghlou);; Ministry of Health and Medical Education, Tehran (S. Mahmoudi, S.M. Zahraei);; Shaheed Beheshti Medical University, Tehran (Z. Chavoshzadeh);; Isfahan University of Medical Sciences School of Medicine, Isfahan, Iran (R. Yazdani);; Children’s Medical Center, Tehran (M. Ebrahimi, M.H. Eslamian)

**Keywords:** poliomyelitis, polio, vaccination, poliovirus, vaccine-derived poliovirus, viruses, children, primary immunodeficiency, Iran

## Abstract

Widespread use of oral poliovirus vaccine has led to an ≈99.9% decrease in global incidence of poliomyelitis (from ≈350,000 cases in 1988 to 74 cases in 2015) and eradication of wild-type poliovirus serotypes 2 and 3. However, patients with primary immunodeficiency might shed vaccine-derived polioviruses (VDPVs) for an extended period, which could pose a major threat to polio eradication programs. Since 1995, sixteen VDPV populations have been isolated from 14 patients with immunodeficiency in Iran. For these patients, vaccine-associated paralysis, mostly in >1 extremity, was the first manifestation of primary immunodeficiency. Seven patients with humoral immunodeficiency cleared VDPV infection more frequently than did 6 patients with combined immunodeficiencies. Our results raise questions about manifestations of VDPVs in immunodeficient patients and the role of cellular immunity against enterovirus infections. On the basis of an association between VDPVs and immunodeficiency, we advocate screening of patients with primary immunodeficiency for shedding of polioviruses.

## Abstract 2

Polio might not be eradicated unless long-term vaccination with inactivated poliovirus vaccine is implemented.

In 1988, the World Health Assembly of the World Health Organization resolved to eradicate poliomyelitis through the introduction of vaccination on a worldwide scale as the main tool against transmission of poliovirus (*1**,**2*). Endemic poliomyelitis has been eliminated from most parts of the world by vaccination with live attenuated oral poliovirus vaccine (OPV) and inactivated poliovirus vaccine (IPV) (*3*). The annual incidence of wild-type poliomyelitis has decreased by ≈99.9% from ≈350,000 cases in 1988 to 74 cases in 2015, and only 2 countries, Afghanistan and Pakistan, still have transmission of wild-type poliovirus (*2*).

As the polio eradication program is proceeding, emergence of vaccine-derived polioviruses (VDPVs) could be a major threat to the success of current strategies. VDPVs are variants of 3 OPV serotypes (PV1, PV2, and PV3), which during prolonged intestinal replication show >1% (PV1 and PV3) or >0.6% (PV2) nucleotide divergence in the viral protein 1 (VP1) coding region (*4*). Only a small number of genetic mutations are responsible for reduced neurovirulence in OPV strains (*5*). Revertant vaccine viruses with increased neuropathogenicity might cause vaccine-associated paralytic poliomyelitis (VAPP) in OPV recipients or unimmunized contacts. VAPP is the most common adverse event after OPV administration (*6*). Because of impaired systemic or mucosal immunity, patients with primary immunodeficiencies are at a markedly increased risk for VAPP (*6*). In addition, patients with primary immunodeficiencies might shed VDPVs (immunodeficiency-associated VDPV [iVDPV]) in stool samples for an extended period after exposure to OPV strains (*7*). Vaccine-derived polioviruses might also circulate in the community (circulating VDPV) through person-to-person transmission and occasionally cause symptomatic infections in immunodeficient or healthy persons (*6*).

In recent years, several studies have focused on environmental detection of VDPVs and their community circulation. Some studies investigated VDPVs in immunodeficient persons, which lead to identification of ≈100 cases of infection with iVDPV worldwide to date (*1**,**8*). We have previously reported several cases of infection with iVDPV in Iran and emphasized the need for surveillance of iVDPV (*9**–**13*). We report newly identified persons who shed iVDPV and review additional characteristics of cases reported.

## Objectives

In this study, we report clinical characteristics of 14 VAPP patients and virologic properties of their iVDPV isolates. All patients were identified in Iran during 1995–2014. We sought to determine relationships between clinical features of underlying immunodeficiency disorders, VDPV manifestations, and patient outcomes.

## Study Design

Patients in Iran with acute flaccid paralysis (AFP) were tested for shedding of polioviruses in stool specimens during national AFP surveillance up to the end of 2014. Stool specimens were collected at the earliest convenience within 48 hours after onset of paralysis. Fecal shedding of vaccine virus strains was simultaneously investigated in close contacts of patients.

Specimens were processed at the Iranian National Polio Laboratory (Tehran, Iran). Virus isolation, serotype identification, and intratypic differentiation were performed by using the World Health Organization protocol for poliovirus detection in stool specimens (*14*). Screening for VDPV shedding was performed by using an ELISA, reverse transcription PCR, or real-time reverse transcription PCR. Genome sequencing of isolates was conducted at the Centers for Disease Control and Prevention (CDC; Atlanta, GA, USA). The VP1 divergence rate was defined as the value (percentage) of VP1 divergence divided by years of virus replication from the original Sabin strain. To calculate this rate, we considered the interval between first OPV administration and date of the most recent isolation of iVDPVs as the duration of virus replication.

Clinical and laboratory data were obtained from the national primary immunodeficiency registry and AFP surveillance database (*15**,**16*). Patients with undetermined immune status were analyzed to determine serum levels of immunoglobulins and absolute leukocyte and different lymphocyte subpopulation counts. Immunity disorders were defined according to the Expert Committee of the International Union of Immunological Societies (*17*). Agammaglobulinemia, hypogammaglobulinemia, and mu heavy chain disease were considered humoral immunodeficiencies. Combined immunodeficiency (CID) included severe combined immunodeficiency, major histocompatibility complex class 2 deficiency, and Nijmegen breakage syndrome.

We evaluated patients by monthly collection of stool specimens and analysis until patients cleared their infections or died of any cause. Patients were classified into separate subgroups according to types of immunodeficiency (humoral or combined) and whether virus shedding in stool specimens was cleared before death. Patients were considered as having cleared an iVDPV infection if 2 consecutive stool specimens were negative for iVDPVs.

## Statistical Analyses

Continuous variables are reported as means ± SD. We used the Shapiro-Wilk normality test to assess distribution of study parameters. We used the χ^2^ test to analyze relationships between categorical parameters, the Mann-Whitney U test to compare duration of shedding and VP1 divergence rate between different subgroups, and an independent *t*-test to compare age of onset of VAPP and absolute VP1 divergence. p values <0.05 were considered statistically significant.

## Results

### Patients and Manifestations

During 1995–2014, a total of 14 patients (12 boys and 2 girls) infected with iVDPV were identified in Iran. Mean ± SD age at onset of VAPP was 12.4 ± 5.8 months for all patients. Mean ± SD age at onset of VAPP was 14.1 ± 4.1 months for patients with humoral immunodeficiency and 10.2 ± 7.5 months for patients with combined immunodeficiency. However, this difference was not significant (p = 0.26). Eight patients had paresis in >1 extremity, and 4 patients had monoparesis. Data were discordant for 2 patients for defining monoparesis or polyparesis.

We analyzed the patients for a mean duration of 18 months; by the end of follow-up, 9 (64.3%) patients had died. As expected, patients with humoral immunodeficiency disorders survived longer than patients with CID (p<0.05). For 5 patients who were alive at the last follow-up (March 2015), mean ± SD age was 4.2 ± 3.3 years. Detailed clinical characteristics for all patients are shown in Table 1, and virologic properties of iVDPVs are shown in Table 2.

**Table 1:** Clinical characteristics for 14 patients infected with iVDPV, Iran, 1995–2014*

Patient no.	Age, mo, at VAPP onset/sex	No. doses OPV	Duration, mo, from last OPV dose to VAPP onset	Duration from VAPP onset to death†	Site of paralysis
1	17.4/F	0	NA	8 d	NA
2	7.5/M	4	1.1	4 mo	Left leg, right leg, right arm, respiratory muscles
3	10.6/M	4	3.3	1 mo	Left leg, right leg, right arm
4	15.1/M	4	9	11 mo	Left leg, right leg
5	5.3/F	2	3.2	<1 mo	Left leg, right leg
6	20.2/M	4	1.1	Alive	Right leg
7	6.2/M	3	2	28 mo	Right leg
8	15.7/M	4	9.2	Alive	Left leg, right leg, left arm
9	25.2/M	4	6.7	<1 mo	Left leg
10	6.6/M	1	6.6	2 mo	Left leg, right leg
11	11.4/M	3	5.3	Alive	Left leg
12	13.1/M	2	7	18 d	Not available
13	10.0/M	4	3	Alive	Left leg, right leg, left arm
14	9.0/M	4	2.8	Alive	Left leg, right leg

*iVDPV, immunodeficiency-associated vaccine-derived poliovirus; NA, not applicable (patient 1 did not receive any OPV): OPV, oral poliovirus vaccine; VAPP, vaccine-associated paralytic poliomyelitis. †Five patients were alive at the most recent follow-up. In March 2015, patient 6 was 9.5 years old, patient 8 was 5 years old, patient 11 was 3.5 years old, and patients 13 and 14 were 1.5 years old.

**Table 2:** Virologic characteristics for 14 patients infected with iVDPV, Iran, 1995–2014*

Patient no.	Report year	Age, mo, at VAPP onset	Virus shedding duration from VAPP onset	iVDPV serotype	VP1 nt divergence, %†	Cleared infection	No. contacts‡
1	1995	17.4	8 d	2	2.2	No	0
2	2005	7.5	3 mo	2	1.5	No	6 (all negative)
3	2006	10.6	2 wk	2	1.7	No	8 (all negative)
4	2006	15.1	5 mo	3	2	Yes	7 (all negative)
5	2007	5.3	5 d	2	2	No	8 (all negative)
5	2007	5.3	5 d	1	1.7	No	8 (all negative)
6	2007	20.2	3 d	2	1.2	Yes	5 (all negative)
7	2011	6.2	15 mo	2	2	Yes	22 (21 negative; 1: P3 SL)
8	2011	15.7	3.5 mo	2	3.8	Yes	6 (4 negative; 1: P1 SL; 1: P1 SL and P2 SL)
9	2011	25.2	4 d	2	3.3	No	6 (all negative)
9	2011	25.2	4 d	1	1.6	No	6 (all negative)
10	2012	6.6	1.5 mo	2	2.3	No	0
11	2012	11.4	2 wk	2	1.5	Yes	6 (all negative)
12	2013	13.1	2 wk	2	0.9	No	4 (3 negative; 1: P1 SL)
13	2014	10.0	2.1 mo	1	1.8	Yes	3 (all negative)
14	2014	9.0	3.3 mo	2	0.6	Yes	3 (all negative)

*iVDPV, immunodeficiency-associated vaccine-derived poliovirus; P1, poliovirus type 1; P2, poliovirus type 2; P3, poliovirus type 3; SL, Sabin-like; VAPP, vaccine-associated paralytic poliomyelitis; VP1, viral protein 1. †Nucleotide divergence from the prototype Sabin strain. ‡No. contacts investigated for VDPVs. Results are indicated in parentheses.

### Types of Primary Immunodeficiencies

Except for 1 patient who died before immunologic studies were completed (patient 12), all patients with VDPV shedding had primary immunodeficiency. Seven patients had humoral immunodeficiencies and 6 patients had CIDs (Table 3). Further investigations and genetic analysis were performed for 8 patients to identify the genes responsible for immunodeficiency. Four patients had mutations in the Bruton tyrosine kinase gene, and a final diagnosis of X-linked agammaglobulinemia was established. Low expression of human leukocyte antigen DR on B lymphocytes indicated major histocompatibility complex class 2 deficiency in patient 2 (*12*). Patient 3 had severe combined immunodeficiency caused by a mutation in recombination activating gene 2 (R229W) (N. Parvaneh, unpub. data). Mutations in Nijmegen breakage syndrome 1 confirmed a diagnosis of Nijmegen breakage syndrome in patient 7, and mu heavy chain genes confirmed mu heavy chain disease in patient 11 (H. Abolhassani, unpub. data).

**Table 3:** Immunologic findings for 14 patients infected with iVDPV, Iran, 1995–2014*

Patient no.	Underlying immunodeficiency	Cells/μL†							Concentration, mg/dL‡		
		Leukocytes	ALC	CD3	CD4	CD8	CD19		IgG	IgM	IgA
1	Undefined hypogammaglobulinemia	NA	NA	NA	NA	NA	NA		NA	NA	NA
2	MHC2	6,300	3,642	1,216	608	607	1,460		200	<10	<10
3	SCID	1,700	731	138	96	32	10		45	<10	<10
4	XLA	6,500	3,375	2,700	1,404	1,290	35		556	<10	<10
5	SCID	6,800	2,589	336	184	185	160		<10	<10	<10
6	XLA	8,500	4,000	2,760	1,920	835	40		20	58	25
7	NBS	6,050	1,040	527	206	264	10		30	22	<10
8	XLA	9,400	4,470	4,201	2,547	1,564	10		80	<10	<10
9	SCID	7,500	3,825	1,092	841	279	2,371		<10	45	<10
10	SCID	7,200	2,174	652	543	163	1,413		40	<10	<10
11	Mu heavy chain	14,400	10,080	8,769	4,636	4,132	110		600§	<10	<10
12	NA¶	NA	NA	NA	NA	NA	NA		NA	NA	NA
13	XLA	10,300	4,738	4,264	3,506	663	30		292	<10	<10
14	Agammaglobulinemia#	10,600	6,254	5,628	4,377	1,375	81		297	34	14

*ALC, absolute lymphocyte count; iVDPV, immunodeficiency-associated vaccine-derived poliovirus; MHC2, major histocompatibility class 2 deficiency; NA, not available; NBS, Nijmegen breakage syndrome; SCID, severe combined immunodeficiency; XLA, X-linked agammaglobulinemia. †Reference range for total leukocytes, 5,000–10,000 cells/μL; reference range for ALC, 3,000–10,800 cells/μL; reference ranges for lymphocyte subpopulations: CD3, 1,900–5,900 cells/μL; CD4, 1,400–4,300 cells/μL; CD8, 500–1,700 cells/μL; CD19, 610–2,600 cells/μL. ‡Reference ranges for serum immunoglobulins: IgG, 246–904 mg/dL; IgM, 40–143 mg/dL; IgA, 27–66 mg/dL. §Patient received intravenous immunoglobulin at a primary medical center before blood sampling was initiated. ¶Not determined because patient died. #Patient was candidate for gene analysis for definitive diagnosis.

### Temporality of VAPP with Diagnosis of Primary Immunodeficiency

Primary immunodeficiencies in patients were detected mostly after onset of paralysis (11 of 14 patients). Only 3 patients had been given a diagnosis of primary immunodeficiency before onset of VAPP. Patient 1 was screened and given a diagnosis of primary immunodeficiency at birth. Patients 9 and 10 received *Mycobacterium bovis* BCG vaccine and a first dose of OPV at birth. These 2 patients had multiple BCG-adenitis and respiratory infections at 10 months and 4 months of age, respectively, which led to a diagnosis of primary immunodeficiency. Despite initiation of immunoglobulin replacement, they subsequently had VAPP.

### VDPV Characteristics in Primary Immunodeficiency Patients

Sixteen iVDPV populations were isolated from the 14 patients; 2 patients were simultaneously shedding iVDPV types 1 and 2. Twelve of 16 viruses isolated were serotype 2, three were serotype 1, and 1 was serotype 3 (Table 2). Overall mean ± SD VP1 divergence was 1.88% ± 0.79%. No differences in values for VP1 divergence were observed between the 2 subgroups of humoral immunodeficiency and combined immunodeficiency patients (p = 0.58). This divergence was also comparable in both subgroups of patients who stopped shedding virus or continued to shed virus (p = 0.75).

The mean ± SD VP1 divergence rate was 1.8% ± 1.1% (range 0.6%–4.3%) per year. Similar to absolute VP1 divergence, the VP1 divergence rate was comparable between 2 subgroups of primary immunodeficiency patients (p = 0.61).

### Shedding Duration and Clearing Infection in Primary Immunodeficiency Categories

Duration of iVDPV isolation for the patients ranged from 3 days to 15 months (median 1 month). Seven patients stopped shedding iVDPV. Seven other patients shed iVDPVs in their last stool specimen before death. If we excluded patient 12, who did not have a definite primary immunodeficiency category, 6 (85.7%) of 7 patients with predominantly humoral immunodeficiencies stopped shedding VDPV, but only 1 (16.7%) of 6 CID patients cleared the infection before death. This association observed between type of primary immunodeficiency and rate of clearing VDPV infection was statistically significant (p = 0.013). Patients with humoral immunodeficiencies cleared VDPV infection more frequently than CID patients (Table 2).

Duration of shedding after VAPP onset was similar in both subgroups of patients with humoral immunodeficiency and combined immunodeficiency (p = 0.66). Analysis also showed a trend of longer shedding duration in patients who stopped shedding iVDPV than in patients who shed the virus until death (p = 0.07).

## Discussion

We analyzed 14 iVDPV patients identified in Iran during 1995−2014. Detailed characteristics of patients 1–7 have been described (*9**–**13*); among these patients, only patient 6 is alive, and this patient has residual paresis. Patient 7, who had AFP at 6 months of age, continued shedding iVDPV2 for 2 months after the most recent published data and then stopped shedding virus. He died at 34 months of age from pneumonia. Among the 7 newly identified case-patients, patient 12 died before he underwent specific immunologic investigations. This patient had a history of recurrent infections, and sequencing data showed quasispecies of VDPV2 from multiple lineages, which suggested immunodeficiency for this patient. This patient was identified as an iVDPV patient in CDC annual reports (*18*).

Serotype 2 iVDPV has been the most prevalent serotype detected in immunodeficient patients (64%), followed by serotypes 1 (21%) and 3 (15%) (*18*); our series showed similar findings. We analyzed 2 patients who were simultaneously shedding serotypes 1 and 2 iVDPVs. Concurrent infection with >1 VDPV serotype has been documented in 3 other patients from China and the United States (*4**,**6**,**19*). Paralysis in poliomyelitis is usually asymmetric (*6**,**20*). In contrast, most of our iVAPP patients had bilateral flaccid paresis. Further studies are required to differentiate manifestations of VDPV infection in primary immunodeficiency patients with poliomyelitis caused by wild-type poliovirus.

Patient 1 received only IPV; however, all his contacts had received OPV as routine national vaccination in Iran. Subsequently, he had VAPP and died of an unknown etiology. All other patients in our study had severe complications of live vaccines (BCG and OPV) given at birth. These complications were the earliest manifestations of primary immunodeficiencies in these patients, which further aggravated their course of disease. Use of screening programs to detect immunodeficiencies at birth could prevent administration of live vaccines to primary immunodeficiency patients and their contacts and minimize the burden of adverse complications.

We observed an association between type of primary immunodeficiency and frequency of clearing iVDPV infection; patients with humoral immunodeficiency cleared VDPV infection more frequently than CID patients. Patients with CID have more severe complications and shorter survival times than patients with humoral immunodeficiency (*16**,**21*). This finding could limit the ability of CID patients to clear infections by use of proper immunoglobulin therapy before death. Although this finding might be an explanation for lower rate of infection clearance in CID patients, comparable durations of virus shedding between 2 subgroups of primary immunodeficiency patients do not support this hypothesis and imply a probable contribution of other mechanisms to this observation.

Another explanation for the relationship observed in our study between type of primary immunodeficiency and rate of clearing iVDPV infection might be differences in inherent capacity of these immunodeficiency disorders to respond to polioviruses or other enterovirus infections. To our knowledge, enteral mucosal immunity against enteroviruses is a prominently antibody-mediated mechanism, and patients with major B-cell dysfunction are at increased risk for poliomyelitis (*6**,**22*). Nevertheless, our observations suggest other major contributions of cellular immunity to clearance of poliovirus infections. The index case-patient for an outbreak of poliovirus infection in Minnesota, USA, in 2005 had been given a diagnosis of severe combined immunodeficiency. She continued to shed iVDPVs while receiving immunoglobulin therapy for several months and finally stopped shedding virus after a second bone marrow transplant (*23*).

Similarly, regular intravenous immunoglobulin (IVIg) administration could not protect patients 9 and 10 in our study from VAPP, and neither patient cleared the infection. Shedding of iVDPV by patient 7 is also notable because he is one of the few case-patients with CID who eliminated the infection before death (*23**,**24*). He had the longest period of iVDPV shedding among our patients before clearing the infection. His duration of virus shedding was several times longer than shedding durations in each of our patients with humoral immunodeficiencies. These observations appear to support the hypothesis of a probable role for cellular immunity in clearing enteroviral infections. Our patients were generally treated with monthly administration of IVIg. However, different IVIg preparations, doses, and settings in immunoglobulin replacement therapy might contribute to efficacy of treatment. Thereafter, to assess the independent role of immune status in clearance of infection, a comprehensive profile of potential confounding factors and therapeutic conditions, which might contribute to capability of virus clearance, is needed. This profile is missing in our study. Additional studies with comprehensive and appropriate adjustments for these factors are required to assess the immunologic response against enteroviral infections in patients with various types of primary immunodeficiencies.

A search for VDPV infection in contacts of our patients yielded negative results, and only Sabin-like polioviruses were isolated from few contacts, which indicates high vaccination coverage in Iran. Spread of iVDPVs to unimmunized contacts in the United States (*6*) and Morocco (*25*) showed potential risk for iVDPV transmission in populations with low immunity, which can lead to an outbreak. Maintaining high vaccination coverage reduces this risk.

The overall rate of accumulative nucleotide substitution in the genome of polioviruses is believed to be ≈1%/year (*6**,**26*). Some investigators consider a range of 1%–2%/year as the overall rate of nucleotide variation in polioviruses (*27**,**28*). Most of our VDPV isolates showed a divergence rate of 1%–2%/year. However, we observed unexpectedly high divergence rates beyond this range in some of our isolates. We propose 3 possible explanations for this difference.

First, for isolates with high divergence rates, the patient could have been infected with an already divergent virus, rather than the original vaccine strain. Thus, the virus had already replicated in a previous host before transmission to the patient. However, lack of any report of circulating VDPV from Iran does not support this explanation. High vaccination coverage and herd immunity against polioviruses in Iran would prevent any circulation of VDPVs. Negative results for VDPV screening in contacts of patients also does not support this explanation.

Second, it has been frequently observed that shortly after OPV administration, the initial rate of capsid region gene evolution is high in Sabin strains, which leads to VDPV emergence (*28**,**29*). A high proportion of these changes are nonsynonymous substitutions in the genomic region, which codes for structural proteins. However, the synonymous mutations, mostly in nonstructural regions, occur at the expected rates. Changes in specific antigenic sites might increase additional nonsynonymous substitutions (*29*). These changes could be caused by effects of direct selection against attenuating mutations in the early stages of VDPV emergence. Selected mutations might carry unselected hitchhiker mutations in these early stages, which would contribute further to a higher initial evolution rate. After these initial selection events, VDPVs appear to evolve at rates closer to the unselected steady-state rate of ≈1%–2%/year. This alternative view is supported by only limited data from more prolonged infections because dates of initiation of most VDPV infections can only be estimated from clinical records but is consistent with available evidence (*27*).

Third, another explanation for higher rates of divergence in some of our isolates could be presence of mixed lineages or quasispecies of VDPVs in stool specimens from corresponding patients. This explanation is consistent with previous evidence, which indicated shedding of mixed-lineage VDPVs by patients with primary immunodeficiency (*27**,**28*). Earlier detection of poliovirus shedding in our patients, possibly after exposure to OPV strains, could have provided precise information about timeline of VP1 divergence in our isolates. This detection could be another advantage of early screening for primary immunodeficiencies and VDPV shedding.

Eradication of wild-type poliovirus serotypes 2 and 3 indicates that eradication of all 3 serotypes can be expected in the near future. However, emergence of VDPVs still remains a threat to global eradication of poliomyelitis (*2*). A review of reports published by CDC showed that ≈100 primary immunodeficiency patients with VDPV shedding have been documented (*1*,*4**,**18**,**19**,**30**–**32*). By the end of 2014, Iran had reported the highest incidence of iVDPV infections (14 case-patients with AFP), followed by the United States (11 case-patients), and the United Kingdom, Egypt, and China (each with 6 case-patients). Similar to the United Kingdom and other industrialized countries, which reported few VAPP cases after switching to vaccination with IPV, only 3 cases occurred in the Unites States after OPV cessation in 2000 (*3**,**23**,**33*). In contrast, the number of iVDPV cases reported from developing and middle-income countries has increased in recent years because of increased extended VDPV surveillance in these countries that used OPV (*18**,**34*).

More than 20 outbreaks of circulating VDPV infection have been documented, mostly in countries with low OPV coverage (*9*). These outbreaks occurred while no cases of infection with iVDPV were reported from some of these countries, such as Nigeria, Democratic Republic of the Congo, Somalia, and Niger, where VDVPs have circulated for a long time (*4**,**9*). To explain this observation, one should consider that patients with primary immunodeficiency do not have a chance for long survival in low-income communities because of lack of proper supportive therapies (*35**,**36*). Thus, even if they are infected with OPV strains, they may die of severe consequences of primary immunodeficiency before emergence of VDPV through prolonged enteral replication.

Environmental isolation of highly divergent VDPVs without an obvious source of shedding has been reported in different countries (*37**–**39*). In Finland, although only IPV vaccine has been administered since 1985, neurovirulent and highly divergent VDPVs have been recurrently detected in sewage since 2008. Epidemiologic data and virologic characteristics suggest that these VDPVs might have originated from chronically infected patients with immunodeficiencies; however, the source of shedding remains to be identified (*38*). Such unidentified chronic shedding of VDPV might reintroduce neurovirulent viruses into the population and initiate outbreaks in the posteradication era if population immunity to poliovirus is not maintained.

High coverage of OPV vaccination disrupted wild-type poliovirus transmission in Iran in the late 20th century, and the last case of wild-type poliomyelitis was detected in 2000 through sensitive AFP surveillance (Figure). Iran has borders with 2 polio-endemic countries (Afghanistan and Pakistan), which necessitates maintaining high levels of serum and mucosal immunity in the population. Although no wild-type poliomyelitis case has been reported from Iran for many years, vaccine-associated paralysis occurs at a rate of a few cases every year and increases illnesses in immunodeficient patients.

**Figure:**
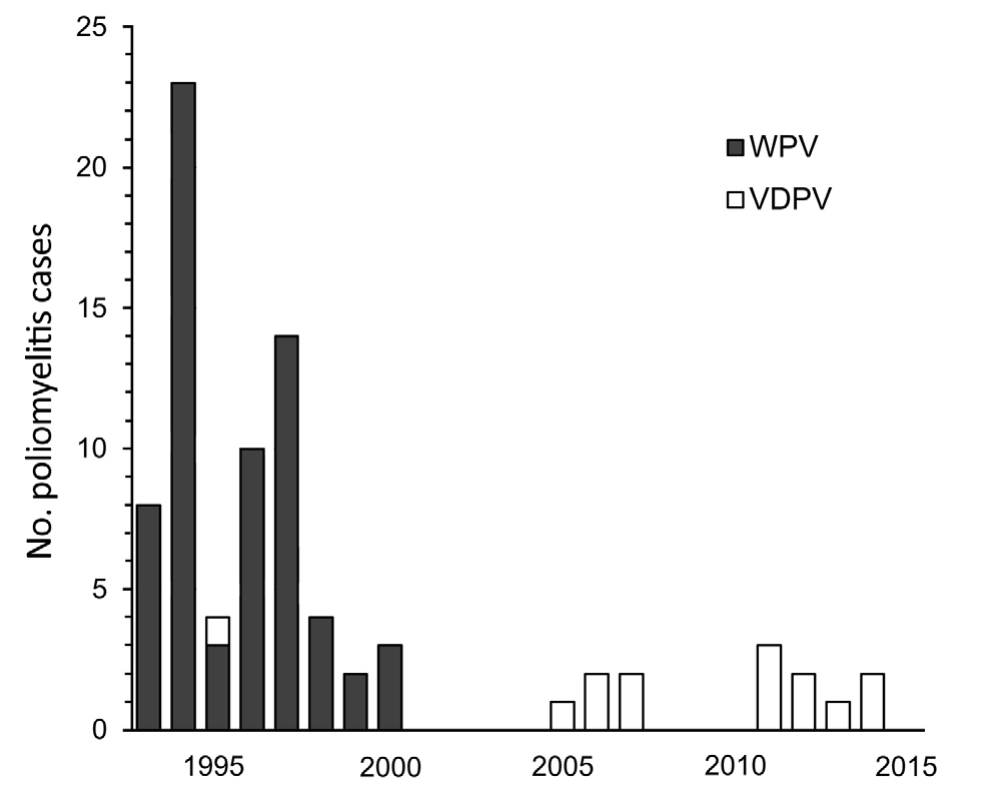
Poliomyelitis cases in Iran, 1995–2014. WPV, wild-type polioviruses; VDPV, vaccine-derived polioviruses.

Routine administration of trivalent OPV would inevitably lead to emergence of VDPVs and new VAPP cases. Conversely, vaccination with IPV might not stop virus transmission because of inefficient induction of mucosal immunity (*40*). However, vaccination with IPV might be too expensive for some low-income countries. Accordingly, the switch to a combination of bivalent OPV (serotypes 1 and 3) and IPV vaccination was implemented worldwide in April 2016 (*2*). We believe that immediate achievement of maximum immunization coverage is another vital point to be addressed in this strategy. Moreover, attempts to develop safer and more efficient poliovirus vaccines and polio antiviral drugs are warranted.

This study highlights the need for efficient iVDPV surveillance and the crucial role of patient registry. Vaccine-derived polioviruses not only endanger countries using OPV but also remain a threat to industrialized countries and the global polio eradication program. Regarding the association of immunodeficiency with this public health issue, we advocate establishment of sensitive clinical and laboratory surveillance to screen primary immunodeficiency patients for shedding of polioviruses.

Eradication might not be accomplished without robust registry and tracking capacities in every country to closely monitor the persistence of polioviruses in the environment and persons at high risk for infection. Achievement of this goal requires providing public health infrastructures, which is missing in some developing countries. In addition, global vigilance of final steps of the eradication program should be increased to attain maximum public contributions. Finally, worldwide IPV immunization should continue with high levels of coverage long after OPV cessation until reliable data indicate worldwide elimination of any poliovirus.
